# Pathological Responses in Asian House Shrews (*Suncus murinus*) to the Naturally Acquired *Orientia tsutsugamushi* Infection

**DOI:** 10.3390/microorganisms12040748

**Published:** 2024-04-07

**Authors:** Tharani Balasubramanian, Uma Sambath, Ranjana Devi Radja, Gowdham Thangaraj, Panneer Devaraju, Lakshmy Srinivasan, Pushpa Srinivasan, Madhavan Gopalakrishnan Nair, Kumar Raja, Avinash Warundeo Lakkawar, Lynn Soong

**Affiliations:** 1 Department of Veterinary Pathology, Rajiv Gandhi Institute of Veterinary Education and Research, Kurumbapet, Puducherry 605009, India; thara6496@gmail.com (T.B.); drsumavet@gmail.com (U.S.); drmgnair@gmail.com (M.G.N.); kumarpath70@gmail.com (K.R.); dralakkawar@yahoo.com (A.W.L.); 2 Unit of One Health, ICMR—Vector Control Research Centre, Indira Nagar, Puducherry 605006, India; ranjanadevi2411@gmail.com (R.D.R.); gowdhamraj1010@gmail.com (G.T.); lakshmysrinivasan1@gmail.com (L.S.); pushakash7@gmail.com (P.S.); 3 Department of Microbiology and Immunology, Institute for Human Infections & Immunity, University of Texas Medical Branch at Galveston, Galveston, TX 77555, USA

**Keywords:** pathological responses, histochemistry, immunohistochemistry, *Orientia tsutsugamushi*, scrub typhus, *Suncus murinus*

## Abstract

Scrub typhus is a re-emerging disease caused by *Orientia tsutsugamushi*, transmitted by mites belonging to the family *Trombiculidae*. Humans and rodents acquire the infection by the bite of larval mites/chiggers. *Suncus murinus*, the Asian house shrew, has been reported to harbor the vector mites and has been naturally infected with *O. tsutsugamushi*. The present study aimed to localize and record *O. tsutsugamushi* in the tissues and the host response in shrews naturally infected with *O. tsutsugamushi*. Sheehan’s modified May–Grunwald Giemsa staining was carried out in 365 tissues from 87 animals, and rickettsiae were documented in 87 tissues from 20 animals. Immunohistochemical (IHC) staining, using polyclonal antibodies raised against selected epitopes of the 56-kDa antigen, was carried out, and 81/87 tissue sections were tested positive for *O. tsutsugamushi*. By IHC, in addition to the endothelium, the pathogen was also demonstrated by IHC in cardiomyocytes, the bronchiolar epithelium, stroma of the lungs, hepatocytes, the bile duct epithelium, the epithelium and goblet cells of intestine, the tubular epithelium of the kidney, and splenic macrophages. Furthermore, the pathogen was confirmed by real-time PCR using blood (*n* = 20) and tissues (*n* = 81) of the IHC-positive animals. None of the blood samples and only 22 out of 81 IHC-positive tissues were tested positive by PCR. By nucleotide sequencing of the 56-kDa gene, Gilliam and Karp strains were found circulating among these animals. Although these bacterial strains are highly virulent and cause a wide range of pathological alterations, hence exploring their adaptive mechanisms of survival in shrews will be of significance. Given that the pathogen localizes in various organs following a transient bacteremia, we recommend the inclusion of tissues from the heart, lung, intestine, and kidney of reservoir animals, in addition to blood samples, for future molecular surveillance of scrub typhus.

## 1. Introduction

Scrub typhus, a febrile illness of substantial incidence and fatality, is caused by an obligate intracellular bacterium, *Orientia tsutsugamushi*. It is estimated that there are more than one million cases that occur every year in the Asia–Pacific region annually, emphasizing the global public health importance of the infection. Scrub typhus is characterized by interstitial pneumonia and, in severe cases, multiorgan system failure. Mites/chiggers belonging to the family Trombiculidae and serve as the vectors and primary reservoirs of the pathogen [1] Transovarian and trans-stadial transmission are the main mechanisms associated with the maintenance of *O. tsutsugamushi* in the vector mites. Humans are accidental hosts; however, rodents and shrews are the main sources of blood meal for the chiggers. Thus, these small mammals play a key role in the enzootic maintenance of the pathogen in nature and transmission of infection to humans. There are no reports of person-to-person transmission of scrub typhus. Outdoor workers, especially field workers in rural areas, have a higher risk of acquiring the disease [1]. The Asian house shrew, *Suncus murinus*, is considered as an indicator host and a major animal reservoir for *O. tsutsugamushi*, especially in Puducherry and other parts of India [2,3]. There is a paucity of information on the tissue response to *O. tsutsugamushi* in the indicator/reservoir hosts, such as shrews (*S. murinus*), that are trapped for surveillance of scrub typhus in endemic areas. The blood collected from these animals is often used for serological or molecular surveillance of scrub typhus. While the pathogen can be detected by molecular methods in the blood of experimentally infected animal models for a limited period of time, it can be identified in various organs over a period of 84 days [4,5,6,7]. In the infected animal models, lesions develop based on the routes of inoculation, failing to establish and reproduce certain pathological features of human scrub typhus [8]. Under natural settings, these small mammals have a similar exposure to the pathogen as humans; however, the disease pathogenesis and response to infection remain obscure. Therefore, this study was carried out to understand the distribution or localization of *O. tsutsugamushi* in the tissues of the natural indicator host (*S. murinus*, Asian house shrews) via histochemical, immunohistochemical, and molecular studies.

## 2. Methods

### 2.1. Trapping and Identification of Shrews and Tissue Sample Collection for Histopathology

This study was carried out in the Union Territory of Puducherry, India. Twenty-three sites were selected randomly for a period of three months (January–March 2020), covering rural, peri-urban, and urban areas to explore the distribution of the reservoirs and vectors of scrub typhus in the Puducherry district. The live trapping of shrews was carried out using Sherman live traps. The approval of the Institutional Animal Ethics Committee of VCRC, Puducherry was obtained for the study (ICMR-VCRC/IAEC/2017/NP-2). Rodent trapping was carried out as per the published procedure of Devaraju et al. (2020) [3]. The traps were set one hour before dusk (17:00 h) and retrieved the next day in the early morning (6:00 h). The trapped live animals were euthanized by exposing them to a chamber saturated with carbon dioxide. Blood from euthanized animals was collected by cardiac puncture. The animals were identified based on their morphological features [9]. The euthanized rodents were dissected, and representative tissue samples from the visceral organs were collected and preserved in 10% neutral buffered formalin (NBF) for histopathological, histochemical, and immunohistochemical studies and in a vial containing PBS for molecular studies, respectively. The animal carcasses were disposed following the norms of the Committee for Control and Supervision of Experiments on Animals. 

### 2.2. Methods Followed for Histology and Special Staining Techniques

Formalin-fixed tissues were processed by a routine paraffin-embedding technique, and 4~5 µm thick sections were prepared and stained by the hematoxylin and eosin (H&E) staining procedure, as previously reported [10]. Following microscopic examination of the H&E-stained tissues and characterization of the changes, representative tissue sections from the various organs were subjected to Giemsa–Sheehan’s modified May–Grunwald staining for the identification of rickettsial organisms, as described by Sheehan and Hrapchak (1980) [11].

### 2.3. Immunohistochemistry (IHC) for Localization of O. tsutsugamushi

Parallel tissue sections, in which rickettsial organisms were detected by Giemsa–Sheehan’s modified May–Grunwald staining technique, were subjected for IHC analysis to assess the cellular distribution of *O. tsutsugamushi* in various organs. The primary antibody was developed by immunizing rabbits with commercially synthesized peptides. The peptides used for immunization were in silico predicted epitopes of the 56-kDa protein of the locally circulating strains of *O. tsutsugamushi.* The details of the peptide sequences are obscured because of patent processing. The IHC procedure involved tissue section preparation, antigen retrieval, blocking of endogenous enzymes, antibody labeling, and visualization.

#### Procedure for Immunohistochemistry

**Figure 1:**
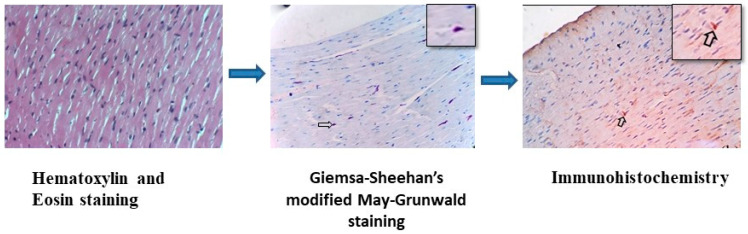
Schematic representation of H&E ×400, Giemsa–Sheehan’s modified May–Grunwald ×400 and IHC immunoperoxidase/DAB substrate/Harris hematoxylin ×400 staining techniques carried out on the heart tissue of *S. murinus*. The purple- and brown-colored cocco-bacillary structures in the insets indicate the *Rickettsia* and *Orientia tsutsugamushi* localized in the tissue by the Giemsa–Sheehan’s modified May–Grunwald and immunohistochemical staining techniques respectively.

Tissue sections were mounted on aminopropyl-triethoxy-silane [APES] (Sigma Chemicals, Rockville, MD, USA)-coated slides and dried at room temperature overnight. The sections were deparaffinized and rehydrated. Antigen retrieval was carried out either by microwaving or by pressure cooking. In the microwave method, the slides were immersed in a citrate buffer (pH 6.0) for 5 min at room temperature and microwaved for 10 min. The slides were then allowed to cool for 20–30 min, rinsed thoroughly in 0.01 M phosphate buffered saline (PBS), and placed in a 0.01 M PBS wash bath for 15 min. Excess fluid from the tissue section was drained out. In the pressure-cooking method, the sides were warmed in a citrate buffer for 5 min, pressure-cooked for 20 min, and then allowed to cool at room temperature for 20 min. The slides were then rinsed thoroughly in 0.01M PBS and placed in a 0.01 M PBS wash bath for 15 min. Excess fluid from the tissue section was drained out. Endogenous peroxidase activity was blocked by covering the whole section with 3% hydrogen peroxide in 0.01 M PBS and incubating at room temperature for 1.5 h. Blocking of the non-specific binding of the antibody was carried out with 5% naïve goat serum for 1 h at room temperature. A polyclonal rabbit anti-*O. tsutsugamushi* antibody (specific to immunodominant epitopes of 56 kDa—outer membrane protein) was used at a dilution of 1:100 for incubation at room temperature for 1 h. After washing, horseradish peroxidase-conjugated goat anti-rabbit IgG-HRP, 1 mL, Genei, Bengaluru, India was used at a dilution of 1:100 and incubated at room temperature for 1 h. Then, 3, 3′ diaminobenzidine tetrahydrochloride (DAB) was freshly prepared for 5–10 min, or until the desired color (light brown) was developed. After washing, nuclear staining was carried out with Harris hematoxylin for 2 min, washed in distilled water, dehydrated, and cleared in xylene. The sections were then mounted with dibutylphthalate polystyrene xylene. The positive immunostaining reaction of the *Orientia* antigens was visualized as brown-colored by the DAB chromogen. Immunohistochemically stained sections were captured using an Optika B5 microscope (Optika, Ponteranica, Italy). The localization of *Orientia* antigens in various tissues was recorded; results were correlated with tissues that were positive for rickettsial organisms by Giemsa–Sheehan’s modified May–Grunwald stain. Figure 1 represents the schematic image of the H&E and Giemsa–Sheehan’s modified May–Grunwald and immunohistochemical staining techniques carried out in the heart tissue of an *S. murinus* that was positive for the *Orientia* antigen. 

### 2.4. Screening O. tsutsugamushi in Blood and Tissues of IHC-Positive Samples by Real-Time PCR and Molecular Typing by Nested PCR 

DNA from the blood and organ samples (collected in PBS) of the animals, which were tested positive for the presence of *O. tsutsugamushi* by IHC staining, was extracted by using Dneasy Blood and Tissue kits (Qiagen, Hilden, Germany). The quality and the quantity of the extracted DNA were analyzed by using a spectrophotometer (Nanodrop, Thermoscientific, Waltham, MA, USA). *O. tsutsugamushi* in the extracted DNA samples was screened by a Taqman qPCR assay targeting the pan-serotype 47-kDa gene [12]. In addition, nested PCR was carried out to amplify the 56-kDa gene of the pathogen [13], and the positive PCR products were nucleotide sequenced. A phylogenetic tree using the 56-kDa nucleotide was constructed by the maximum likelihood method with 1000 bootstrap replicates on the Mega X platform to identify the clustering of the replicates into various serotypes. 

## 3. Results 

### 3.1. Histochemical Staining for Rickettsial Organisms in Various Tissues of S. murinus

**Figure 2:**
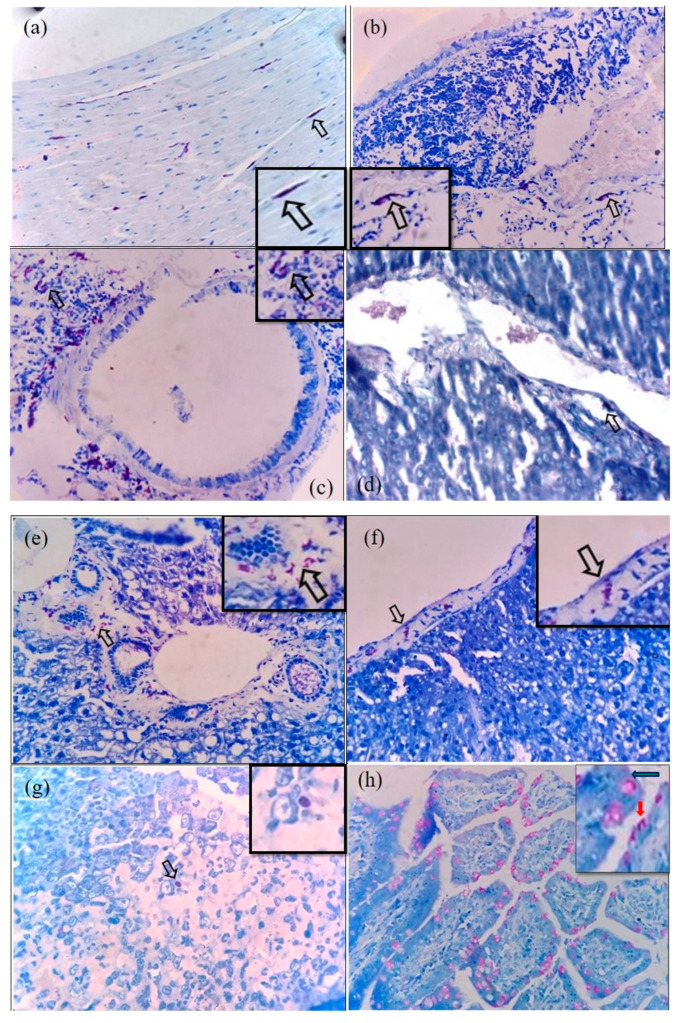

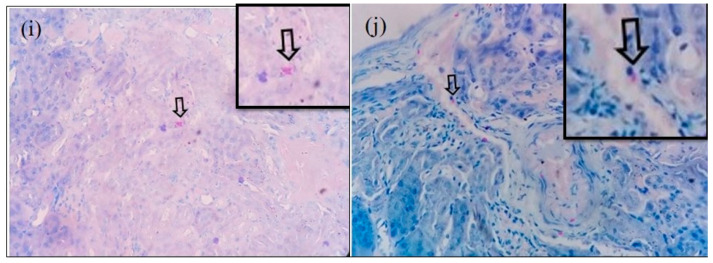
Representative heart tissue samples of shrews stained positive for Rickettsia by Giemsa–Sheehan’s modified May–Grunwald stain appearing as reddish-purple-color in the endothelium of blood vessels and in the cytoplasm of cardiomyocytes ×400 (**a**); endothelial cells of pulmonary blood vessels ×400 (**b**); in peribronchiolar infiltrates in lung ×400 (**c**); endothelial cells of central vein of liver ×400 (**d**); in stromal cells of portal triad ×400 (**e**); Glisson’s capsule ×400 (**f**); in splenic macrophages ×400 (**g**); intestinal epithelial cells and goblet cells ×400 (**h**); in renal tubular epithelium ×400 (**i**) and endothelial cells of renal blood vessels ×400 (**j**). Insets shows digitally enhanced views of the original image (×400) to indicate the rickettsial organims.

By Giemsa–Sheehan’s modified May–Grunwald staining method, the rickettsial agents localized in the tissues appeared as reddish-purple-colored round-to-coccobacillary organisms varying from 0.5 to 1.0 µm in size. Our representative images of *Rickettsia*-positive tissues are shown in Figure 2a–i. In most of the tissues, the rickettsial organisms were detected in the endothelium of blood vessels either singly or in clusters. Additionally, in some of the tissues (liver, lung, and spleen), the organisms could be identified in the cytoplasm of neutrophils and macrophages. Out of 87 animals screened, rickettsial organisms were detected in the tissues from 20 animals. These included the heart (*n* = 20), lung (*n* = 15), liver (*n* = 20), spleen (*n* = 15), intestine (*n* = 15), and kidneys (*n* = 2). All the tissue samples (*n* = 87) from the 20 animals that were positive for rickettsial organisms by histochemical methods were subjected to immunohistochemistry.

### 3.2. Immunohistochemical Detection of O. tsutsugamushi in the Tissues of S. murinus

**Figure 3:**
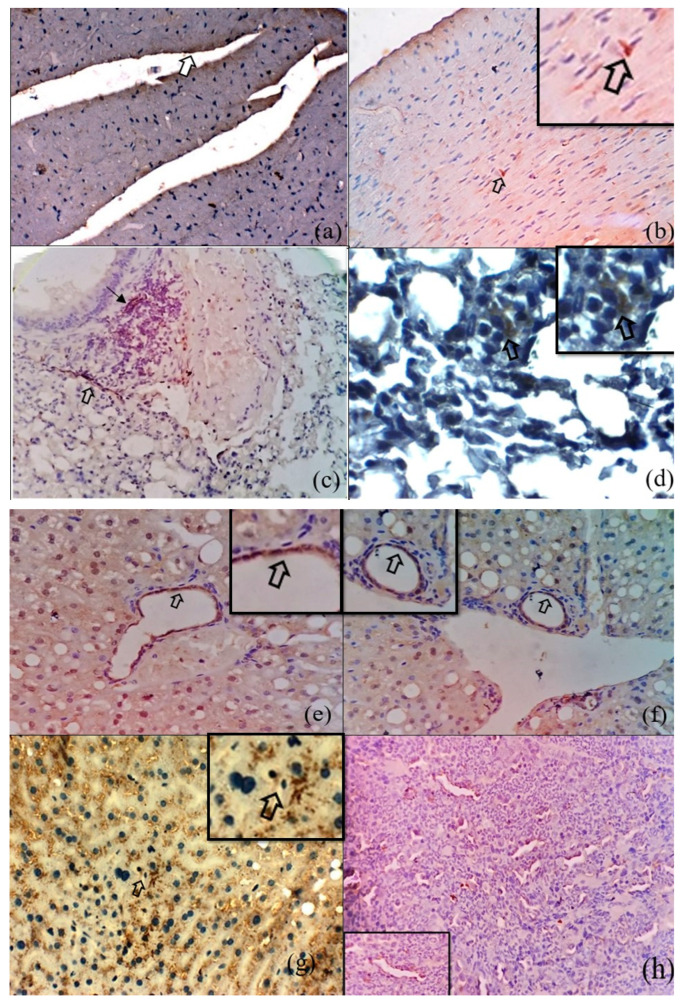

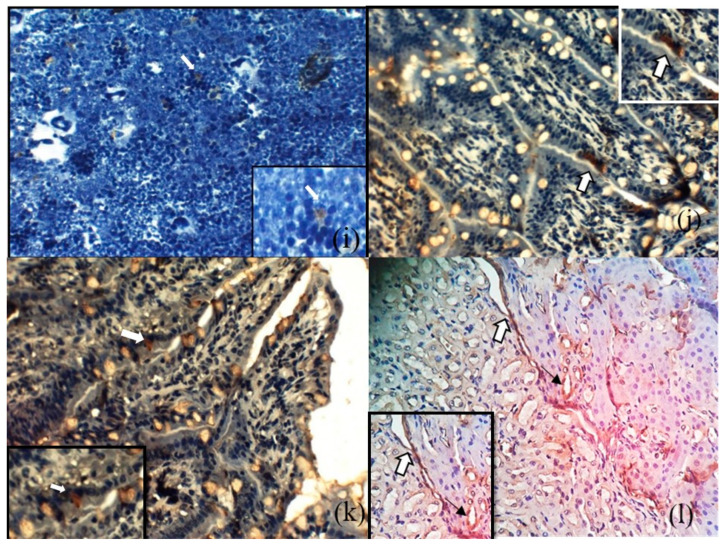
Representative images of the IHC positive immunostaining (IHC immunoperoxidase/DAB substrate/Harris hematoxylin) of Orientia antigen, visualized as brown-color in the endothelium of endocardium ×400 (**a**); in cardiomyocytes (inset) ×400 (**b**); endothelium of pulmonary blood vessel (open arrow) and in peribronchiolar infiltrates ×400 (arrow) (**c**) and within alveolar macrophages ×1000 (inset) (**d**); in liver—endothelium of central vein ×200 (inset) (**e**), in bile duct epithelium ×200 (inset) (**f**) and within hepatocytes ×400 (inset) (**g**); in splenic sinusoids ×200 (inset) (**h**) and in splenic macrophages ×200 (inset) (**i**); in intestinal epithelial cell ×400 (inset) (**j**,**k**); in renal tubular epithelium (arrow) and in endothelium of renal blood vessels (open arrow) ×200 (inset) (**l**).

**Figure 4:**
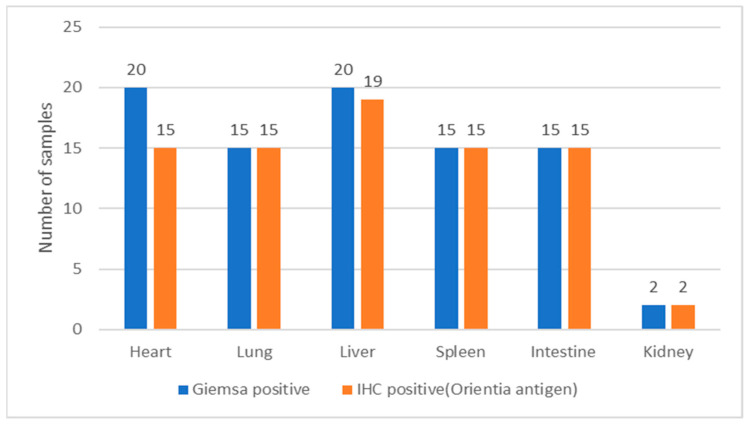
Comparison of histochemical and IHC staining for localization of Rickettsia/*Orientia* in the tissues of *Suncus murinus*.

By IHC staining, 81 tissue samples were positive for *O. tsutsugamushi* 56-kDa antigens; these included 15 heart, 15 lung, 19 liver, 15 spleen, 15 intestine, and 2 kidney samples. Representative images of IHC-stained tissues are shown in Figure 3a–l. In general, the *O. tsutsugamushi* antigens were detected in the endothelial cells of the blood vessels of the tissues examined. Additionally, the organisms were localized in the cardiomyocytes, bronchiolar epithelium, alveolar septa and interstitial capillary vessels, peribronchiolar infiltrate, hepatocytes and bile duct epithelium, and intestinal epithelial cells, including goblet cells, the renal tubular epithelium, and splenic macrophages. The Figure 4, illustrates the comparison of Rickettsia/Orientia localization in the tissues of Suncus murinus by histochemical and IHC staining methods respectively.

### 3.3. Microscopic Features of Orientia-Antigen-Positive Tissues 

Table 1 and Table 2 summarizes the microscopic features of various *Orientia*-positive tissues via histochemical and IHC staining techniques. Of the 15 heart tissues, normal histological features were observed in 13 samples. The histopathological changes recorded in the two heart samples included mild vacuolar degeneration, focal areas of necrosis, linear hemorrhages, and fibrosis. In the 15 lung samples, normal histological features were observed in 3 samples. The pathological findings observed in the twelve samples were congestion, hemorrhage, emphysematous changes, and neutrophil infiltration. Other findings in the lung samples were mononuclear cell infiltration, pyogranuloma, focal areas of necrosis, and hemosiderin pigmentation. The *Orientia*-antigen-positive liver tissues (*n* = 20) showed mostly cell swelling, fatty changes, and vacuolar degeneration. Other lesions observed were amyloidosis, congestion, karyomegaly, neutrophil, mononuclear cell and mixed cell infiltration, fibrosis, and extramedullary hematopoiesis. The histopathological examination of 15 spleen samples revealed normal histological features in 6 samples. Hyperplasia of white pulp was the predominant change observed, and the other lesions observed were hyperplasia of red and white pulp, atrophy of white pulp, and pigmentation. In *Orientia*-antigen-positive intestine tissues (*n* = 15), normal histological features were observed in 12 samples. The histopathological lesions noticed were hyperplasia of the intestinal epithelium and parasitic enteritis. The *Orientia* antigen was detected in two kidney samples that had normal histological features. Of note, 36/81 (44.4%) of tissues that were positive for the *Orientia* antigen had normal histological features, and 35/81 (43.2%) had degenerative or inflammatory lesions. However, 10/81 (12.3%) animals exhibited other lesions, including hemosiderin pigmentation and inflammatory responses to parasites such as *Capillaria* sp. and *Hepatozoon* sp. 

#### Molecular Detection of *O. tsutsugamushi* in Blood and Tissue Samples

A total of 20 blood samples (corresponding to the IHC-positive samples) and 81 tissue samples that tested positive by IHC were screened for *O. tsutsugamushi* by qPCR. None of the blood samples were tested positive for the presence of the pathogen by qPCR. We observed that only 22 (27%) out of 81 tissue samples tested positive for *O. tsutsugamushi* by qPCR. The details of PCR positivity in the tissue samples screened are given in Table 3. 

**Figure 5:**
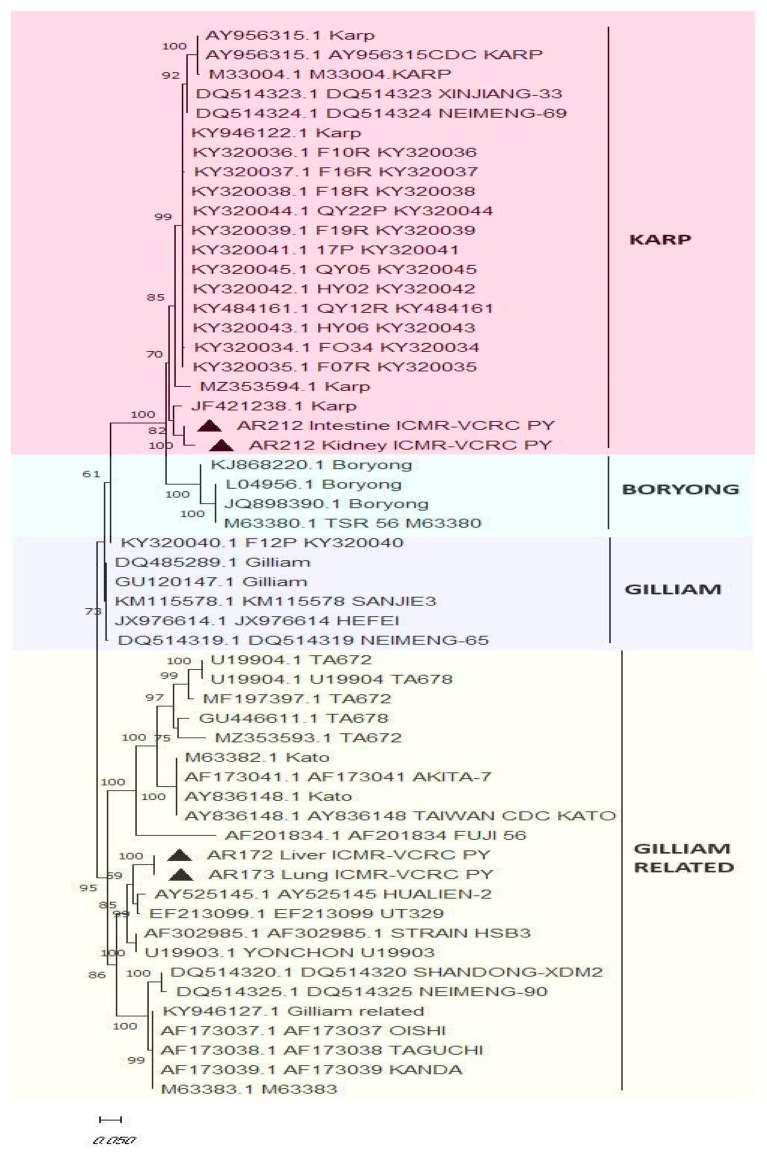
Phylogenetic clustering of the isolates of *Orientia tsutsugamushi* based on the 56-kDa nucleotide sequences. The phylogenetic tree was constructed using the maximum likelihood method with 1000 bootstrap replicates on Mega X platform. The solid triangle symbol indicates the isolates from the current study.

Amplification of the 56-kDa gene by nested PCR was successful in 4/22 samples tested positive by qPCR. It was observed that the DNA derived from the intestine and kidney of the same animal and a lung and liver sample from two animals also tested positive for *O. tsutsugamushi* by nested PCR. A phylogenetic tree constructed using the nucleotide sequences of the 56-kDa gene revealed that the isolates clustered with the sequences of Gilliam and Karp (Figure 5), the two major strains known to be endemic in our studied sites. 

## 4. Discussion

Scrub typhus is a re-emerging infectious disease of major public health importance that is endemic throughout India, with seasonal upsurges of cases during cooler months of the year [14]. Though the natural course of the infection in humans is well studied, the exact pathogenesis of the disease in humans could not be replicated in animal models. The Asian house shrew, *S. murinus*, the indicator host for scrub typhus, acquires the infection naturally, but the information on the pathology caused by, *O. tsutsugamushi* in various organ systems of shrews has so far not been reported. Therefore, the objective of the present study was to localize *O. tsutsugamushi* and to study the pathological responses in tissues of free-ranging/wild *S. murinus*. 

Experimental animal studies on the *O. tsutsugamushi* Karp strain have been carried out using various routes of inoculation to record the localization of bacteria in tissues [5,6,7]. Kundin et al. (1964) found the antigen to be distributed in an inoculation-route-specific manner [15]. In the intraperitoneally inoculated animals, the *Orientia* antigen was observed predominantly on the peritoneal surface of the liver and spleen, which does not resemble the clinical human disease and histopathology. Upon inoculation of *O. tsutsugamushi* by an intravenous route, the *Orientia* antigen was predominantly located in the endothelial cells of the lung, kidney, and liver at the early stages [7]. Later, with disease progression, the organism was detected in all tissues, resulting in hematogenously disseminated endothelial infection, which mimicked human disease but bypassed the events of early cutaneous infection and the initial dissemination [8]. Since natural human infections are initiated via mites feeding on the dermis of the skin, Shelite et al. (2014) developed an intra-dermal inoculation model of scrub typhus that developed a systemic infection with body temperature changes, weight loss, and bacterial dissemination via the blood to other major organs [8] and closely mimicked the course of infection observed in human scrub typhus [16]. By experimental footpad inoculation, which combined both intradermal and subcutaneous routes, Kamala et al. (2007) and Long et al. (2013) reported that the *Orientia* antigen initially localizes in the regional lymph node and eventually spreads predominantly to the lungs and heart [17,18]. However, in our study, the shrews that acquired the pathogen naturally through the bites of the infected mites were studied for histological changes. In general, *O. tsutsugamushi* antigens are detected in the endothelial cells of the blood vessels of all the organs studied, which concurs with the findings in animals experimentally infected with *O. tsutsugamushi* [5,6,7,8,14,15,16,17]. 

In the present study, we followed Giemsa–Sheehan’s modified May–Grunwald method to demonstrate rickettsial organisms in the tissue sections (*n* = 365) from the organs of 87 *S. murinus.* Out of 87 animals screened, rickettsial organisms were detected in the tissues from 20 animals. Rickettsial organisms were detected in the endothelium of blood vessels in most of the tissues. Additionally, in some of the tissues (liver, lung, and spleen), the organisms could be identified in the cytoplasm of neutrophils and macrophages. 

The common H&E histologic findings in scrub typhus are vasculitis, dilation of the capillary blood vessels, and infiltration of monocytes in the vicinity of capillaries. Yet, such findings cannot be used as diagnostic features for scrub typhus, as the above-mentioned histopathological features are commonly shared by the other infectious agents. Hence, scrub typhus should be definitively diagnosed by assessing and localizing *Orientia tsutsugamushi* in the tissues by IHC. For rickettsioses such as rickettsialpox and Rocky Mountain spotted fever, the diagnostic usefulness of immunohistochemical staining has been reported, and immunohistochemical staining can be applied as the definitive diagnostic method [19].

All the tissue samples (*n* = 87) from the 20 animals that tested positive for rickettsial organisms by histochemical methods were subjected to immunohistochemistry. A good correlation was observed between the results of histochemical studies and the immunohistochemistry findings. However, six tissue specimens that tested positive for rickettsiae by the special staining method tested negative by IHC, which could be due to infection by other natural rickettsial agents of the shrews. Kuo et al. (2015) reported the presence of *R. japonica*, *R. rickettsii*, *Rickettsia* sp. TwKM01, and *R. typhi* in shrews trapped in Taiwan. They reported that 50% of the trapped shrews tested positive for either SFG or TG and 8.3% of the animals had mixed infection [20]. Lu et al. (2019) reported the presence of the *R. parkeri*-like strain, *R. raoultii*, *A. phagocytophilum*, *Ehrlichia* sp., and *Candidatus Neoehrlichia mikurensis* in rodents and shrews trapped from Ganzhou, China [21]. The above two studies indicate that the shrews could naturally harbor diverse rickettsial agents. The identity of the rickettsial agents other than *O. tsutsugamushi* was not explored in our study. 

Interestingly, our findings of the endotheliotropism of *O. tsutsugamushi* in various organs of shrews are in line with the IHC studies carried out in humans by Moron et al. (2001) in archived autopsy tissues samples (heart, lung, kidney, pancreas, and skin) of soldiers with scrub typhus infection [22]. Demonstration of the organism in the cardiac tissue in the shrews in our study is in tandem with the reports in human infections by Moron et al. (2001) [22] and Tseng et al. (2008) [23]. The major difference is that the polyclonal antibodies raised against the whole pathogen were used by them, whereas we employed the polyclonal antibodies raised against the selected immunodominant epitopes of the 56-kDa antigen. The 56-kDa protein is a key molecule for cellular adhesion and intracellular penetration of *O. tsutsugamushi* into host cells. This protein is abundant in the outer membrane of the pathogen, constitutes 10–15% of the total rickettsial cellular protein content, and has been reported to be highly immunogenic [24,25].

IHC staining of the shrew specimens detected *Orientia* in the endothelial cells of all the organs evaluated, including many blood vessels without perivascular mononuclear infiltrates. But, in the present study, in addition to the localization of *O. tsutsugamushi* in the endothelium of alveolar and interstitial capillaries, the *Orientia* antigen was also noticed in the peribronchiolar infiltrates in 15 out of 15 samples. We have also additionally demonstrated the localization of the pathogen in cardiomyocytes, the bronchiolar epithelium, alveolar septal cells, interstitial capillary vessels, peribronchiolar infiltrate, splenic macrophages, and intestinal epithelial cells, including goblet cells and the renal tubular epithelium. 

Moron et al. (2001) carried out anti-*Orientia* IHC studies to show the bacteria in macrophages within mouse tissue sections following infection with Karp and Gilliam strains [22]. The rickettsiae were located within macrophages that had infiltrated the capsules of the liver and spleen, as well as in splenic macrophages. Moron et al. (2001) [22] and Tseng et al. (2008) [23] localized *O. tsutsugamushi* within macrophages of the liver. In the present study, *Orientia* was detected in the endothelium of blood vessels, bile duct epithelium, and cytoplasm of hepatocytes in 18 out of 20 liver samples. The observations in our study also indicated the presence of *O. tsutsugamushi* in the macrophages of the spleen in 8 out of 20 samples. Mostly, *O. tsutsugamushi* was in the endothelium of sinusoids in the splenic parenchyma, as in the observations of Moron et al. (2001) [22]. Tseng et al. (2008) [23] found *O. tsutsugamushi* in the endothelial cells of the appendix; this was the first report to describe this organism in the gastrointestinal tract. In the present study, *O. tsutsugamushi* was also immunolocalized in the intestinal epithelial cells in eight out of eight samples and within the goblet cells in two out of eight samples. 

The *O. tsutsugamushi* antigen was also detected in the renal tubular epithelium and endothelium of blood vessels in one sample from *S. murinus*. Tseng et al. (2008) [23] reported that, in addition to endothelial cells, *O. tsutsugamushi* may be distributed in the reticuloendothelial system, such as macrophages of the liver, spleen, lymph node, and bone marrow, suggesting that *O. tsutsugamushi* may disseminate into multiple visceral organs via endothelial cells and macrophages, resulting in fatal complications in human cases.

None of the blood samples from the 20 IHC-positive animals tested positive for *O. tsutsugamushi* by PCR, indicating that these animals have crossed the window of rickettsemia in the host. However, PCR positivity observed in the heart, lung, liver, spleen, kidney, and intestine indicates that these animals have acquired infection and have had a dissemination of the pathogen to various organ systems. Soong et al. (2016) [6] reported the undetectable, detectable, and peak durations of *O. tsutsugamushi* in various tissues of experimentally infected C57BL/6 mice models. They reported that the pathogen was detectable at 9 days post inoculation (d.p.i) and peaked at 15 d.p.i in the blood. Sustained detection at lower levels was reported in tissues such as spleen, brain, and liver from 13 d.p.i until 70 and 63 d.p.i, respectively. However, the pathogen was detectable by PCR in the kidney until 70–77 d.p.i. The differences in the positivity obtained between PCR and IHC assays could be due to the detection limits of PCR in relation to the availability of an adequate quantity of gene copies. 

In our previous study, we observed PCR negativity for *Orientia tsutsugamushi* in the blood of the trapped animals, which could be due to transient rickettsemia. Also, in the same animals, PCR screening of organs such as the heart, lungs, intestine, and kidney yielded positive results [26]. In the present study, by using Giemsa–Sheehan’s modified May–Grunwald staining and immunohistochemistry techniques, we demonstrated the localization of *Orientia tsutsugamushi* in various tissues of the above animals. Hence, based on the concordance of the findings between the two studies and persistence of the pathogen in the tissues despite transient rickettsemia, it is suggested that organs such as the heart, lungs, liver, spleen, and intestine could also be considered for the zoonotic surveillance of *Orientia tsutsugamushi* in animal reservoirs.

Compared to other laboratory animals, the spontaneous pathology due to *O. tsutsugamushi* in the Asian house shrew (*S. murinus*) is obscure. Histopathological examination of various tissues from *S. murinus*, in the present study, primarily trapped for screening *O. tsutsugamushi*, had certain limitations, such as details of the age, virulence of the pathogen, previous history of exposure, seroconversion, and disease duration in the infected animals. However, this study has revealed the spectrum of host responses in the form of pathological lesions, affecting the various organ systems under natural exposure to the pathogen. Among the IHC positivity for *O. tsutsugamushi* in various tissues, 68% of heart, 20% of lung, 2.35% of liver, 37.1% of spleen, 51.5% of intestine, and all of the kidney tissues exhibited normal histological features. This indicates that the shrews have a strong innate immune response against the pathogen. This aspect, however, needs to be further investigated in shrews. 

Lethality to the Karp strain of *O. tsutsugamushi* has been reported in outbred CD-1 mice, and many of the studies have used a less pathogenic Gilliam strain in experimental mice models, such as C57BL/6 [6], CD-1 [27] and humanized DRAGA mice [28]. However, 56-kDa gene sequencing and analysis revealed the circulation of the Gilliam, Karp [2,3], and Kato [3]-like strains among the trapped shrews in Puducherry [2,3]. Demonstration of lethal strains like Karp in these animals, trapped alive from natural settings, indicates that they could tolerate virulent strains. Hence, further studies are required to demonstrate the virulence of various strains of *O. tsutsugamushi* in natural reservoirs under laboratory conditions. These findings would help to understand species-specific immune responses to various strains of *O. tsutsugamushi*, which would contribute to designing an effective vaccine against scrub typhus. 

## 5. Conclusions

Through histochemical and IHC studies, we found that the antigens of *O. tsutsugamushi*, the causative agent of scrub typhus, could be demonstrated in various tissues of Asian shrew (*Suncus murinus*) with varied pathological responses. Future investigation into biological mechanisms behind the survival of these animals (despite being infected by the virulent pathogenic strains) would provide clues for developing treatment or prophylactic measures. For future molecular surveillance of *O. tsutsugamushi* in index animals (such as rodents and shrews), we recommend the inclusion of tissues from the lungs, heart, and liver, in addition to blood samples, in order to enhance the sensitivity of detection. 

## Figures and Tables

**Table 1:** Summary of microscopic features recorded in *Orientia*-antigen-positive tissues.

S.no.	Organs	Features	Frequency of Lesions
1	Heart	Normal histology	13
		Vacuolar degeneration	1
		Focal necrosis	1
		Neutrophil infiltration	1
		Linear hemorrhage	1
		Fibrosis	1
2	Lung	Normal histology	3
		Congestion	9
		Hemorrhage	5
		Emphysema	4
		Neutrophil infiltration	6
		Mononuclear infiltration	1
		Pyogranuloma	1
		Focal necrosis	1
		Pigmentation	1
3	Liver	Cell swelling	12
		Vacuolar degeneration	2
		Fatty change	13
		Karyomegaly	1
		Neutrophil infiltration	3
		Mononuclear infiltration	3
		Mixed cell infiltration	1
		Congestion	2
		Fibrosis	2
		Extramedullary hematopoiesis	1
4	Spleen	Normal histology	6
		Hyperplasia of white pulp	3
		Hyperplasia of red pulp	3
		Atrophy of white pulp	1
		Pigmentation	1
		Neutrophil infiltration	1
5	Intestine	Normal histology	12
		Hyperplasia of intestinal epithelium	3
		Parasitic enteritis	3
6	Kidney	Normal histology	2

**Table 2:** Consolidation of microscopic features in various tissues of *Suncus murinus* that were positive for *Orientia* antigen.

S.no.	Features	No.
1	Number of tissues immunopositive for *O. tsutsugamushi*	81
2	Number of *O. tsutsugamushi* immunopositive tissues with normal histological features	36
3	Number of *O. tsutsugamushi* immunopositive tissues with degenerative/inflammatory lesions	35
4	Number of *O. tsutsugamushi* immunopositive tissues with other histopathological changes (vascular changes, pigmentation, growth disturbances)	10

**Table 3:** Results of real-time PCR carried out on various tissue samples tested positive for *O. tsutsugamushi* by IHC.

Sl.No	Tissues Tested Positive for *O. tsutsugamushi* by PCR						Total
	Heart	Lung	Liver	Spleen	Intestine	Kidney	
No. of samples tested positive for *O. tsutsugamushi* by IHC	19	15	15	15	15	2	81
No. of samples tested positive for *O. tsutsugamushi* by real-time PCR targeting the 47-kDa gene	5	9	3	1	3	1	22

## Data Availability

The data pertaining to this manuscript will be shared upon reasonable request to the corresponding author.
